# Tightly Coupled GNSS/IMU Hybrid Navigation Using Factor Graph Optimization with NLOS Detection Capability

**DOI:** 10.3390/s26072264

**Published:** 2026-04-06

**Authors:** Haruki Tanimura, Toshiaki Tsujii

**Affiliations:** Department of Aerospace Engineering, Graduate School of Engineering, Osaka Metropolitan University, Nakamozu Campus, Osaka 599-8531, Japan; harukit.1158@gmail.com

**Keywords:** GNSS, machine learning, NLOS, IMU, factor graph optimization, tightly coupled, urban environment, positioning accuracy

## Abstract

High-precision and reliable self-localization is essential for autonomous navigation systems. However, in urban canyons (urban environments with clusters of high-rise buildings), Global Navigation Satellite Systems (GNSS) suffer from severe multipath and Non-Line-of-Sight (NLOS) signal reception. This causes a theoretically unbounded positive bias in pseudorange measurements, significantly degrading positioning integrity. To address this challenge, this study proposes a novel GNSS/Inertial Measurement Unit (IMU) tightly coupled integrated navigation system using factor graph optimization (FGO) integrated with machine learning-based NLOS detection. To train the NLOS detection model, we utilized a dual-polarized antenna to label signals based on the strength difference between RHCP and LHCP components, achieving a detection accuracy of 0.89. A random forest classifier identifies NLOS signals, and based on its classification labels, the variance of the corresponding GNSS pseudorange factors within the FGO framework is dynamically inflated. This effectively mitigates the impact of outliers while preserving the graph topology. Experimental evaluations in dense urban environments demonstrated that the proposed method improves horizontal positioning accuracy by 84.8% compared to conventional standalone GNSS positioning. The dynamic integration of machine learning-based signal classification and tightly coupled FGO provides an extremely robust positioning solution, proven to meet the stringent reliability requirements demanded of autonomous systems even under severe signal obscuration.

## 1. Introduction

The societal implementation of next-generation mobility systems, including autonomous vehicles and unmanned aerial vehicles (UAVs), heavily relies on the accuracy of self-localization systems. Maintaining lane-level control in advanced driver assistance systems (ADAS) and autonomous driving generally requires sub-meter-level positioning accuracy (e.g., below 0.5 m) [1,2]. However, meeting these stringent requirements remains a significant challenge in dense urban environments known as urban canyons. Building obstructions cause GNSS signals to reflect and diffract, frequently resulting in Non-Line-of-Sight (NLOS) reception where direct waves (LOS) are completely blocked and only reflected waves are received. NLOS signals distort the correlation waveform within the receiver, introducing a positive bias on pseudo-range measurements that can range from tens to hundreds of meters with theoretically no upper limit, ultimately causing the positioning solution to fail fundamentally [3,4,5].

To compensate for the vulnerability of standalone GNSS positioning, integrated navigation systems combining GNSS with an inertial measurement unit (IMU) are standard practice [6,7]. Traditionally, the Extended Kalman Filter (EKF) has been widely adopted for this sensor fusion. However, the EKF assumes Markovian properties, performing sequential estimation by marginalizing past states into a single covariance matrix. This structural limitation causes the EKF to suffer from problems such as high linearization error accumulation when initial state uncertainty is large or under the strong nonlinear conditions characteristic of urban environments [8]. As a robust alternative, factor graph optimization (FGO) has garnered significant attention in recent years [9]. FGO enables batch processing over a fixed period of past observations (a sliding window) and performs iterative relinearization (using methods like Gauss–Newton or Levenberg–Marquardt) across the entire state trajectory. This mechanism effectively utilizes temporal correlations between observations, enabling more reliable convergence to optimal estimates and demonstrating excellent robustness against non-Gaussian outliers [8,9]. Specifically, the tightly coupled (TC) FGO, which directly integrates raw GNSS observations (such as pseudorange) with the IMU’s pre-integration factor, is an extremely advantageous method in urban areas because it can utilize GNSS updates even in environments with fewer than four visible satellites [10].

Although FGO possesses excellent optimization capabilities, indiscriminately incorporating extreme NLOS bias into the graph as a uniform Gaussian noise model causes the optimization solver to attempt fitting to erroneous observations, inevitably leading the estimated trajectory to deviate from the true value. Therefore, preprocessing to detect NLOS signals and appropriately eliminate or mitigate their effects is essential before executing the optimization algorithm. GNSS signals are inherently transmitted as right-hand circularly polarized (RHCP). However, when physically reflected off surfaces like building walls, their phase inverts, resulting in a dominant left-hand circularly polarized (LHCP) component. Recent studies have demonstrated the effectiveness of utilizing the signal strength difference between RHCP and LHCP signals obtained from dual-polarization antennas as an indicator for NLOS detection, leveraging this physical characteristic [11]. Furthermore, to address complex signal propagation characteristics, machine learning (ML) approaches such as random forests are being actively researched. These methods learn nonlinear decision boundaries using multiple features, such as satellite elevation angle, signal strength, and polarization intensity difference, enabling high-precision classification of LOS/NLOS signals [12,13].

However, a comprehensive approach to dynamically and seamlessly integrate these advanced machine learning-based NLOS classification results into the graph structure of TC FGO, thereby enhancing the overall system robustness, remains insufficiently established. Therefore, this study proposes a novel GNSS/IMU tightly coupled integrated navigation system equipped with machine learning-based NLOS detection capabilities. This method uses a random forest model to evaluate signal features and classify the NLOS state of each satellite signal. Based on the inferred labels, it dynamically adjusts the variance (weight) of the corresponding GNSS pseudorange factor within the TC FGO framework. By assigning extremely large variances to factors classified as NLOS, this method effectively neutralizes the detrimental effects of outliers in the optimization process while maintaining the structural connectivity of the graph. Through real-world driving experiments in severe multipath environments, we quantitatively verify how the proposed method significantly suppresses positioning errors and achieves stable continuous navigation even under conditions of partial GNSS signal loss.

This paper is structured as follows: Section 1 outlines the research background and objectives; Section 2 details machine learning-based NLOS detection; Section 3 details tightly coupled GNSS/IMU integration using TC FGO; Section 4 details the positioning experiment; and Section 5 summarizes the findings and discusses future work.

## 2. Machine Learning-Based NLOS Detection

**Figure 1:**
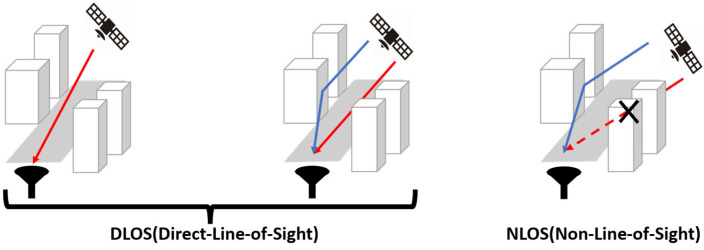
Classification of GNSS signals. The red arrow indicates a direct signal, and the blue arrow indicates a reflected signal.

GNSS signals can be broadly categorized into three signal types (Figure 1): LOS (Line-of-Sight), which receives only direct signals; Multipath, which receives both direct and reflected signals; and NLOS (Non-Line-of-Sight), which receives only reflected signals. Multipath and NLOS signals, which include reflected signals, contain errors and are generally not recommended for use in positioning.

In NLOS environments where the direct signal is not received, the upper limit of the error is infinite, often leading to large errors. Therefore, excluding NLOS satellites from positioning is expected to improve positioning accuracy.

We collectively refer to satellites where the direct signal is received (LOS) and those with multipath (Multipath) as DLOS (direct-line-of-sight). We then detected the NLOS satellites most affecting positioning using machine learning.

In this study, we constructed an NLOS detection model using the random forest machine learning algorithm [14]. Random forest is an ensemble learning method combining multiple decision trees, aggregating the prediction results of each tree via majority vote to generate the final decision. For training the decision trees, data is randomly sampled with replacement from the entire dataset. Furthermore, the features used for node splitting within the decision trees are also randomly selected. This inherent randomness helps prevent overfitting, where the model becomes overly dependent on the training data. By combining different trees, the model achieves extremely high prediction accuracy. An additional advantage is that the classification process is not a black box; it allows for the quantification of the importance of each feature.

In creating an NLOS detection model using machine learning, it is necessary to classify training data into NLOS and DLOS. This study performed this labeling by utilizing the characteristics of GNSS signals. Signals from GNSS satellites arrive as right-hand circularly polarized (RHCP) waves, propagating in a clockwise spiral. However, when these signals reflect off surfaces like building walls, the left-hand circularly polarized (LHCP) component, which spirals counterclockwise, tends to dominate. This study focuses on the signal strength of data acquired from each antenna element—RHCP and LHCP—using a dual circularly polarized antenna. When the direct wave is received, the signal strength acquired by the RHCP element is expected to be greater than that acquired by the LHCP element, and vice versa when only the reflected wave is received. Therefore, by taking the difference between the RHCP and LHCP signal strengths for each satellite signal, the signal type can be classified and labeled. Let the RHCP and LHCP signal strengths be $C/N_{0,RHCP}^{\left(i\right)}$ and $C/N_{0,LHCP}^{\left(i\right)}$, respectively. Define their difference as in Equation (1).

(1) $$∆C/N_{0}^{\left(i\right)}=C/N_{0,RHCP}^{\left(i\right)}-C/N_{0,LHCP}^{\left(i\right)},$$

Using this, define the reception state $St_{i}$ of satellite i as in Equation (2).

(2) $$St_{i}=\{\begin{array}{c} DLOS,\;\;\;if\;∆C/N_{0}^{\left(i\right)}\geq 0\\ NLOS,\;\;\;if\;∆C/N_{0}^{\left(i\right)}<0 \end{array},$$

Specifically, if the difference between the RHCP and LHCP signal strengths is positive, it is considered DLOS; if negative, it is considered NLOS.

**Figure 2:**
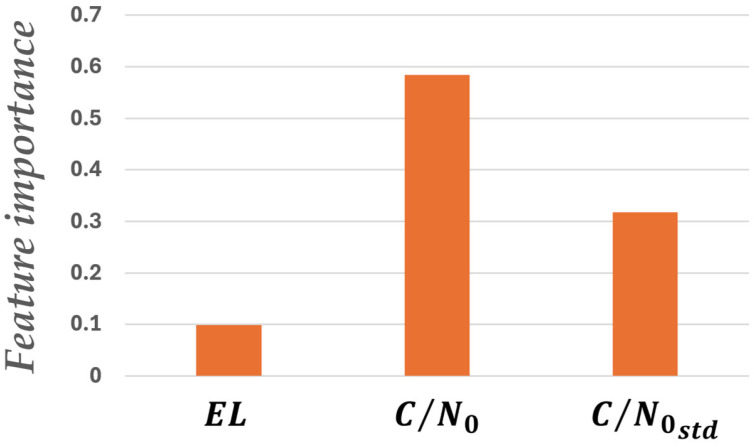
Feature importance. $C/N_{0}$ contributes most to signal classification.

In this study, signals received from GNSS satellites were classified as NLOS or DLOS. Satellite elevation angle, $C/N_{0}$, and the standard deviation of $C/N_{0}$ were used for this classification, as shown in Table 1 [12]. Satellite elevation angle represents the angle of the satellite relative to the receiving antenna. Generally, a lower satellite elevation angle increases the likelihood of receiving reflected signals from obstacles such as buildings. The carrier-to-noise density ratio ($C/N_{0}$) is obtained as the ratio of signal power to noise power density. Typically, signals attenuate due to reflection, so a lower value suggests a higher probability of NLOS conditions. The standard deviation of $C/N_{0}$ is calculated using data from a total of 100 epochs (the current data point and the preceding 99 consecutive epochs). Generally, greater signal strength variation is considered indicative of a higher probability of NLOS. Figure 2 shows the importance of the three features used in this study. It was found that $C/N_{0}$ contributes most significantly to classification.

**Figure 3:**
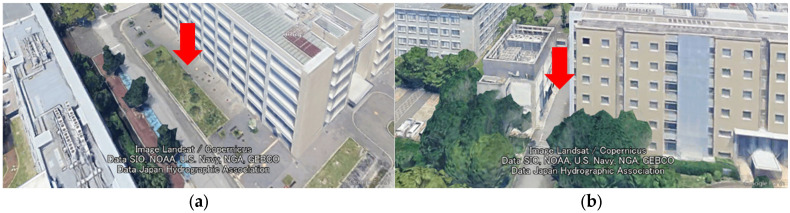
Data acquisition environment for training data generation. (**a**) A location surrounded by buildings. (**b**) A location surrounded by buildings (similar to (**a**)). (Image: Google Earth Pro, © 2026 Google.)

**Figure 4:**
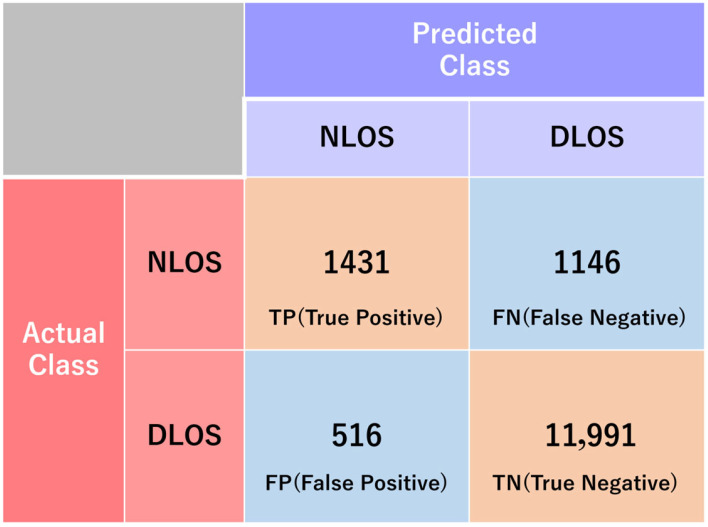
Confusion matrix. Here, NLOS is considered positive.

Based on the above method, an NLOS detection model was constructed. At the two locations shown in Figure 3, data was acquired for one hour at a 1 Hz data rate using a dual circularly polarized antenna. Subsequently, signals were labeled as NLOS or DLOS based on RHCP and LHCP signal strengths. Eighty percent of the total data was used for model training, and the remaining 20% was used for testing. The test results are shown in Figure 4, achieving an accuracy of 0.89, confirming the model’s usefulness for classification. The accuracy (ACC) is calculated using the classification results shown in Figure 4 and Equation (3). By using the NLOS detection model created in this way, the final system does not require dual-polarization antennas and can be applied to data acquired using a single-polarization antenna.

(3) $$ACC=\frac{TP+TN}{TP+TN+FP+FN},$$

## 3. Tightly Coupled GNSS/IMU Integration Using FGO

### 3.1. Overview and Features of FGO

In practical navigation systems, it is anticipated that various sensors will be integrated alongside GNSS. This study implemented a GNSS/IMU integrated navigation system using FGO (factor graph optimization). In recent years, FGO has gained attention in the field of multi-sensor fusion as an alternative approach to conventional filtering methods. FGO is a method that formulates the optimization problem in multi-sensor fusion using probabilistic graphical models. Compared to the Extended Kalman Filter (EKF), which has been widely used in GNSS/IMU integrated navigation, FGO has the following characteristics.

First, it utilizes historical data through batch processing. While the EKF assumes Markovian properties and compresses past information into a covariance matrix for recursive estimation, FGO retains all past observations and optimizes all states simultaneously. This allows for more effective utilization of temporal correlations between observations in environments where GNSS signals degrade, such as urban areas, thereby improving robustness against outliers.

Second, it employs iterative linearization. The EKF performs linearization only once per time step. Consequently, if the initial estimate error is large, linearization errors accumulate. In contrast, FGO employs iterative optimization using methods like Gauss–Newton or Levenberg–Marquardt. Each iteration involves relinearization, enabling FGO to converge more readily to optimal estimates even when initial errors are significant.

Third is flexibility through graph structure. FGO represents the system state at each time step as a “node” and observations or constraints as “factors” within a graph structure. Adding new factors to this graph structure allows for the incorporation of new sensor observations or constraint conditions, offering high system extensibility.

This research aimed to improve positioning accuracy and achieve robust navigation in poor signal reception environments by implementing GNSS/IMU integrated navigation using FGO, which possesses the above advantages.

GNSS/IMU integrated navigation using FGO primarily employs two methods: the loosely coupled method (hereafter referred to as LC FGO) and the tightly coupled method (hereafter referred to as TC FGO). A key difference between them lies in the type of GNSS data used for integration. In LC FGO, the receiver position estimated internally by the GNSS receiver is directly integrated. While this method is relatively easy to implement, it requires the GNSS receiver to acquire signals from at least four satellites to calculate a positioning solution. In contrast, TC FGO uses raw GNSS observations such as pseudorange. This method allows the use of observations as update information even when fewer than four satellites are observable. Consequently, it is considered more robust than LC FGO in environments where GNSS signal reception deteriorates, such as urban areas. This study implemented GNSS/IMU integrated navigation using TC FGO to leverage its robustness in urban environments.

### 3.2. FGO’s Algorithm

FGO is a method that formulates the multi-sensor integration problem as a probabilistic graphical model and defines it as a Maximum A Posteriori (MAP) estimation problem that maximizes the posterior probability of the entire state. Assuming that the observations from GNSS and IMU are mutually independent, the optimal state estimate $\hat{X}$ is obtained by maximizing the joint probability distribution as shown in Equation (4).

(4) $$\hat{X}=argmax\prod_{k,i}P(z_{k,i}|x_{k})\prod_{k}P(x_{k}|x_{k-1},u_{k}),$$

Here, $z_{k,i}$ represents the i-th observation value (e.g., GNSS pseudorange) at time k, $u_{k}$ denotes the control input (IMU observation), and $x_{k}$ represents the system state to be estimated. In FGO, the above probabilistic model is represented using a factor graph. Each probability term in Equation (4) is treated as a factor $\phi_{j}$ connecting nodes on the graph. Therefore, the MAP estimation problem is formulated as maximizing the product of all factors, as shown in Equation (5).

(5) $$\hat{X}=argmax\left(\prod_{j}\phi_{j}\left(x_{j}\right)\right),$$

Here, we introduce the assumption that all sensor noise and process noise follow a Gaussian distribution. Since the probability density function of a Gaussian distribution with mean μ and covariance matrix Σ takes the form of an exponential function, each factor $\phi_{j}$ is defined using the observed value $z_{j}$ and the observation function $h_{j}(x_{j})$ as shown in Equation (6).

(6) $$\phi_{j}\left(x_{j}\right)\propto \exp\left(-\frac{1}{2}{\left(h_{j}\left(x_{j}\right)-z_{j}\right)}^{T}\Sigma_{j}^{-1}\left(h_{j}\left(x_{j}\right)-z_{j}\right)\right),$$

The exponential term in Equation (6) corresponds to the squared Mahalanobis distance ${\left\|h_{j}\left(x_{j}\right)-z_{j}\right\|}_{\Sigma_{j}}^{2}$. Therefore, the intensity of a factor is proportional to the square of the error multiplied by a negative exponent. To transform the product form into a sum form, we take the negative log-likelihood of the objective function. Since the logarithm function is monotonically increasing, maximizing the original probability product is equivalent to minimizing the negative logarithm, as shown in Equation (7).

(7) $$\begin{array}{ll} \hat{X} & =argmin(-\ln(\underset{j}{\prod }\phi_{j}\left(x_{j}\right)))\\ & =argmin\underset{j}{\sum }\left(-\ln\phi_{j}\left(x_{j}\right)\right) \end{array}$$

Substituting the factor definition based on the Gaussian distribution here cancels out the natural logarithm and exponential functions, ultimately yielding the nonlinear least squares problem expressed by Equation (8).

(8) $$\hat{X}=argmin\sum_{j}{\left\|h_{j}\left(x_{j}\right)-z_{j}\right\|}_{\Sigma_{j}}^{2},$$

As described above, FGO enables the simultaneous optimization of all states using an iterative optimization algorithm by reducing the probabilistic estimation problem to a weighted least squares minimization problem.

### 3.3. Tightly Coupled GNSS/IMU Integration Using FGO

**Figure 5:**
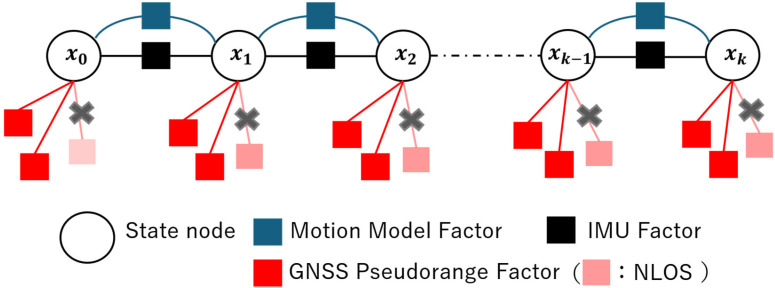
Graph structure. Construct a graph structure connecting state nodes and factors. NLOS signals are effectively ignored (as explained in detail in Section 3.4).

This section describes the specific formulation of the GNSS/IMU fusion using TC FGO [8]. In TC FGO, the raw GNSS observations (pseudorange) and IMU observations are integrated on a common graph structure, and the entire state is optimized (Figure 5). Since TC FGO directly handles GNSS observations, it requires estimating the receiver clock error in addition to the cases for LC FGO. The system state vector $x_{k}$ at time k is defined as in Equation (9).

(9) $$x_{k}={\left(X_{k}^{ecef},V_{k}^{ecef},B_{k}^{body},\delta_{{clock}_{k}}\right)}^{T},$$

Here, the definitions of each variable in Equation (9) are as follows.

- $X_{k}^{ecef}$: receiver position (x,y,z);
- $V_{k}^{ecef}$: receiver velocity $\left(v_{x},v_{y},v_{z}\right)$;
- $B_{k}^{body}$: acceleration bias of the IMU $\left(b_{x},b_{y},b_{z}\right)$;
- $\delta_{{clock}_{k}}$: receiver clock bias.

The optimal state set $X^{*}=\left\{x_{1},\ldots ,x_{k},\ldots \right\}$ in TC FGO is obtained by minimizing the sum of squared errors (sum of Mahalanobis distances) for all factors within the graph. The objective function is expressed as in Equation (10).

(10) $$X^{*}=argmin(\sum_{k}{\left\|e_{{GNSS}_{k}}\right\|}_{\Sigma_{{SV}_{k}}}^{2}+\sum_{k}{\left\|e_{{MM}_{k}}\right\|}_{\Sigma_{{MM}_{k}}}^{2}+\sum_{k}{\left\|e_{{IMU}_{k}}\right\|}_{\Sigma_{{IMU}_{k}}}^{2}),$$

Equation (10) consists of three terms: the GNSS pseudorange factor, the motion model factor, and the IMU factor. Details of each factor are described below.

#### 3.3.1. GNSS Pseudorange Factor

This factor is based on the pseudorange from each satellite. Let $\rho_{SV}$ denote the observed pseudorange vector of all satellites at time k. The error function $e_{{GNSS}_{k}}$ is defined as the residual between the observed value and the theoretical pseudorange calculated from the estimated state position, as shown in Equation (11) below.

(11) $${\left\|e_{{GNSS}_{k}}\right\|}_{\Sigma_{{SV}_{k}}}^{2}={\left\|\rho_{SV}-h_{GNSS,TC}(x_{k})\right\|}_{\Sigma_{{SV}_{k}}}^{2},$$

Here, the i-th element of the observation function $h_{GNSS,TC}$ is defined as the geometric distance between the known satellite position $SV_{k,i}$ and the estimated receiver position $X_{k}^{ecef}$, plus the receiver clock error $\delta_{{clock}_{k}}$, and is expressed by Equation (12).

(12) $$h_{GNSS,TC,i}\left(\cdot \right)=\left\|SV_{k,i}-X_{k}^{ecef}\right\|+\delta_{{clock}_{k}},$$

Furthermore, the covariance matrix $\Sigma_{{SV}_{k,i}}$ is configured to perform weighting based on the reliability of observations, which is determined by each satellite’s signal strength ($C/N_{0}$) and elevation angle. This capability for adaptive weighting according to the signal quality from each satellite is a key feature of TC FGO. In this study, the covariance matrix $\Sigma_{SV_{k}}$ for the GNSS pseudorange factor is defined as a diagonal matrix with variance $\sigma_{i}^{2}$ in its diagonal elements, as shown in Equation (13).

(13) $$\begin{array}{cc} \Sigma_{SV_{k}} & =diag\left(\sigma_{1}^{2},\ldots ,\sigma_{N}^{2}\right)\\ \sigma_{i}^{2} & =\frac{1}{\sin^{2}el_{i}}\times \left(10^{-\frac{{C/N_{0}}_{i}-T}{a}}((\frac{A}{10^{-\frac{F-T}{a}}}-1)\frac{{C/N_{0}}_{i}-T}{F-T}+1)\right) \end{array}$$

Here, the definitions of each variable and constant are as follows.

- $el_{i}$: elevation angle of satellite i;
- ${C/N_{0}}_{i}$: signal strength of satellite i;
- a, A, F, T: constant parameter (a=30, A=30, F=10, T=45).

In other words, the lower the satellite elevation angle and the weaker the signal strength, the larger the variance $\sigma^{2}$ becomes, resulting in a smaller weight in the optimization process.

#### 3.3.2. Motion Model Factor

The motion model factor serves as a constraint to maintain physical consistency in state transitions between two consecutive time points, k−1 and k. In this study, a uniform motion model is employed, and the error function takes the form given by Equation (14).

(14) $${\left\|e_{{MM}_{k}}\right\|}_{\Sigma_{{MM}_{k}}}^{2}={\left\|{\left(X_{k}^{ecef},B_{k}^{body},\delta_{{clock}_{k}}\right)}^{T}-h_{MM}(x_{k-1})\right\|}_{\Sigma_{{MM}_{k}}}^{2},$$

Here, the function $h_{MM}(\cdot )$ functions as a state transition matrix, specifically describing the physical relationships between states as in Equation (15).

(15) $$h_{MM}\left(x_{k-1}\right)=\left[\begin{array}{c} X_{k-1}^{ecef}+V_{k-1}^{ecef}\cdot ∆t\\ B_{k-1}^{body}\\ \delta_{{clock}_{k-1}} \end{array}\right],$$

Furthermore, the covariance matrix $\Sigma_{{MM}_{k}}$ represents process noise. In this study, it is represented as a constant diagonal matrix in Equation (16).

(16) $$\Sigma_{{MM}_{k}}=diag(1.0^{2},1.0^{2},1.0^{2},0.01^{2},0.01^{2},0.01^{2},10^{2}),$$

Here, the first three components represent position-related noise, the next three components represent acceleration bias-related noise, and the final component represents receiver clock error-related noise.

#### 3.3.3. IMU Factor

The IMU factor serves as a control input to incorporate high-rate acceleration data from the IMU into the system’s state update. In this study, IMU data is used solely for velocity constraints. Since the raw acceleration $A_{raw}$ output by the IMU is in the sensor coordinate system, it is transformed to the Earth-fixed coordinate system based on the definition of the state vector. Using attitude information obtained from the AHRS (Attitude and Heading Reference System), the transformation is performed according to Equation (17).

(17) $$A_{k}^{ecef}=R_{GL}R_{LB}(A_{raw,k}-B_{k}^{body}),$$

Here, the definitions of each matrix are as follows.

- $B_{k}^{body}$: accelerometer bias;
- $R_{LB}$: rotation matrix from sensor coordinate system to ENU coordinate system;
- $R_{GL}$: rotation matrix from the ENU coordinate system to the ECEF coordinate system.

Using the transformed acceleration $A_{k}^{ecef}$, the error function is expressed as in Equation (18).

(18) $${\left\|e_{{IMU}_{k}}\right\|}_{\Sigma_{{IMU}_{k}}}^{2}={\left\|V_{k}^{ecef}-h_{IMU}(x_{k-1},A_{k}^{ecef})\right\|}_{\Sigma_{IMU_{k}}}^{2},$$

Here, the observation function $h_{IMU}(\cdot )$ predicts the current velocity by adding the integral value of acceleration to the previous time’s velocity, as shown in Equation (19).

(19) $$h_{IMU}(x_{k-1},A_{k}^{ecef})=\left[V_{k-1}^{ecef}+A_{k}^{ecef}\cdot ∆t\right],$$

Furthermore, the covariance matrix $\Sigma_{IMU_{k}}$ represents the measurement noise of the IMU’s acceleration sensor. In this study, it is expressed as a constant diagonal matrix in Equation (20).

(20) $$\Sigma_{IMU_{k}}=diag(0.01^{2},0.01^{2},0.01^{2}),$$

Equation (10), the objective function derived from the formulation of the above three factors, is a nonlinear least-squares problem. Therefore, it cannot be solved analytically, and the optimal solution was numerically searched using an iterative solution method called the Levenberg–Marquardt method [15].

### 3.4. Integration with NLOS Detection Function

**Figure 6:**
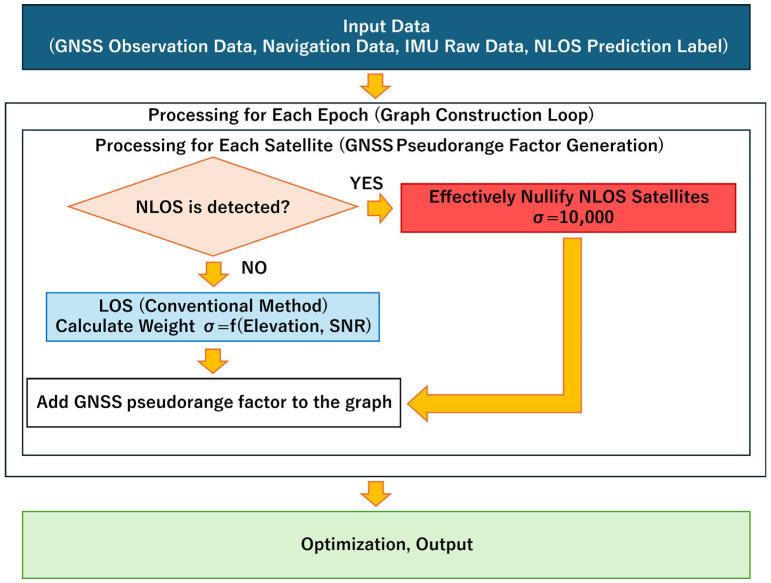
Workflow for analysis processing with integrated NLOS detection.

In the TC FGO algorithm described in the preceding sections, a uniform noise model based on Equation (13) was applied to all satellite signals. This model assumes that observation errors follow a zero-mean Gaussian distribution. However, in urban areas, the reception of NLOS signals is expected to result in a non-Gaussian distribution with a long tail. To address this situation, this study employs a method that dynamically switches the uncertainty of the observed GNSS pseudorange values based on the NLOS determination results obtained in advance using the machine learning model detailed in Section 2. Figure 6 shows the analytical processing flow of the proposed method.

As input to this system, in addition to GNSS observation data and IMU acceleration data, CSV-formatted NLOS prediction labels are used, which have been pre-determined based on the reception status of each satellite. The determination label for satellite i at time k is defined as $L_{k,i}$ and expressed as in Equation (21).

(21) $$L_{k,i}\in \left\{0:DLOS,1:NLOS\right\},$$

Label information read from CSV files is stored in a map structure and referenced during graph construction for each epoch. In the conventional TC FGO, a noise model expressed by Equation (13) was applied to all satellites. However, this implementation dynamically switches the applied noise model based on the value of label $L_{k,i}$.

When $L_{k,i}=0$, the corresponding satellite signal is considered valid, so the weighted model given by Equation (13) is applied. This allows for the maximum utilization of high-quality satellite signal information, thereby improving estimation accuracy.

When $L_{k,i}=1$, the corresponding satellite signal is highly likely to contain significant bias due to reflections from buildings, etc., necessitating measures to prevent adverse effects on optimization calculations. In this implementation, to eliminate the influence of such observations while preserving graph connectivity, an extremely large variance as described in Equation (22) below shall be assigned to them.

(22) $$\sigma_{k,i}^{2}=\sigma_{penalty}^{2}=10000^{2},$$

In the least squares problem within FGO, the error term is multiplied by the inverse of the variance. Consequently, this setting renders the weight of the corresponding factor extremely small, effectively nullifying its contribution to the optimization results.

By implementing dynamic switching of the noise model based on the above NLOS determination results, the error function for the GNSS pseudorange factor in the TC FGO of the proposed method is defined as in Equation (23).

(23) $${\left\|e_{{GNSS}_{k}}\right\|}_{\Sigma_{{SV}_{k}}}^{2}\;\;\;\;\;where\;\;\;\;\;\Sigma_{{SV}_{k}}=\{\begin{array}{c} diag\left(\sigma_{k,i}^{2}\right)\\ diag(10000^{2}) \end{array}\;\;\;\;\;\begin{array}{c} if\;L_{k,i}=0\\ if\;L_{k,i}=1 \end{array},$$

## 4. Positioning Experiment

### 4.1. Experimental Setup and Methods

**Figure 7:**
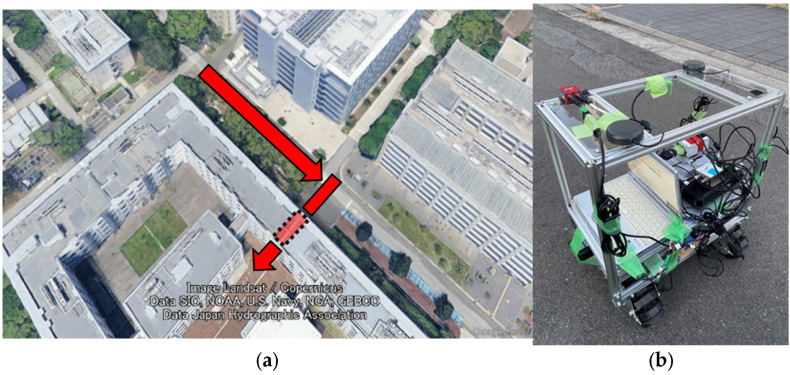
(**a**) Experimental environment. The red arrow indicates the rover’s path. The section enclosed by the black dotted line is the portion that passes beneath the building (image: Google Earth Pro, © 2026 Google); (**b**) the rover used in the experiment.

**Figure 8:**
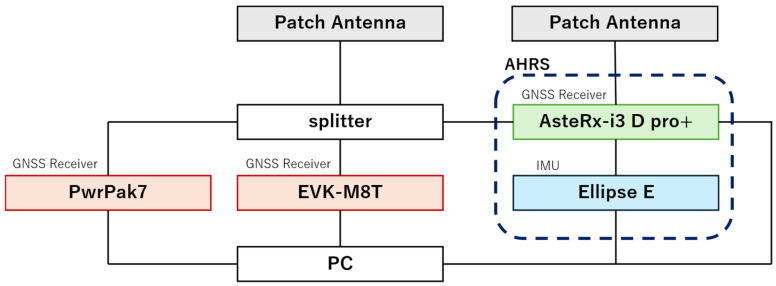
Connection diagram of equipment installed on the rover.

We implemented a GNSS/IMU integrated navigation system using TC FGO with integrated Non-Line-of-Sight (NLOS) detection functionality as the proposed method. We conducted mobile experiments to evaluate positioning errors, robustness, and system effectiveness. Figure 7a shows the experimental environment. The route traversed an area surrounded by high-rise buildings, passing beneath the building cluster to simulate a poor GNSS signal reception environment. The section enclosed by the black dotted line in Figure 7a is the portion that passes beneath the building. A rover (Figure 7b) was used as the mobile platform and operated remotely. Figure 8 shows the connection diagram of the equipment installed on the rover. Two patch antennas (Calian Inc., Ottawa, ON, Canada: TW7972) were used to acquire GNSS signals. Three GNSS receivers were installed: (ublox, Tharwil, Switzerland: EVK-M8T), (NovAtel, Calgary, AB, Canada: PwrPak7), and (Septentrio, Leuven, Belgium: AsteRx-i3 D pro+). The EVK-M8T acquired GPS, Galileo, and QZSS data at a 1 Hz data rate and was used for evaluation. The IMU (SBG, Carrières-sur-Seine, France: Ellipse-E) acquired data at 200 Hz. Two GNSS antennas, the AsteRx-i3 D pro+ and the Ellipse-E, were used for attitude angle estimation in the AHRS. By establishing a reference station, the GNSS-RTK solution from the GNSS receiver (NovAtel, Calgary, AB, Canada: PwrPak7) was used as the reference value for error comparison. However, during sections where GNSS signals were blocked while passing under buildings, the reference value was partially generated using linear interpolation. RTKLIB ver 2.4.2 [16] was used to compute both the standalone GNSS positioning solution and the relative positioning solution.

In this experiment, we compared the following three methods.

1. GNSS-only positioning

A standard single-point positioning solution obtained by analyzing data from a GNSS receiver. This method directly experiences the effects of NLOS and thus serves as a baseline for evaluation.

2. TC FGO-Based GNSS/IMU Integrated Navigation

The method described in the previous section employs Equation (13) as the weighting factor in the TC FGO algorithm and utilizes all observed satellite signals. While improved accuracy through integration with IMU data is expected, the approach may be susceptible to non-Gaussian observation errors caused by NLOS effects.

3. Proposed Method: GNSS/IMU Integrated Navigation Using TC FGO with Integrated NLOS Detection

TC FGO is integrated with dynamic NLOS determination using the machine learning-based NLOS detection model described in Section 2. For satellites determined to be NLOS, an extremely large variance is assigned (Equation (22)) to maintain DOP (Dilution of Precision) while nullifying their influence.

### 4.2. Results and Analysis

**Figure 9:**
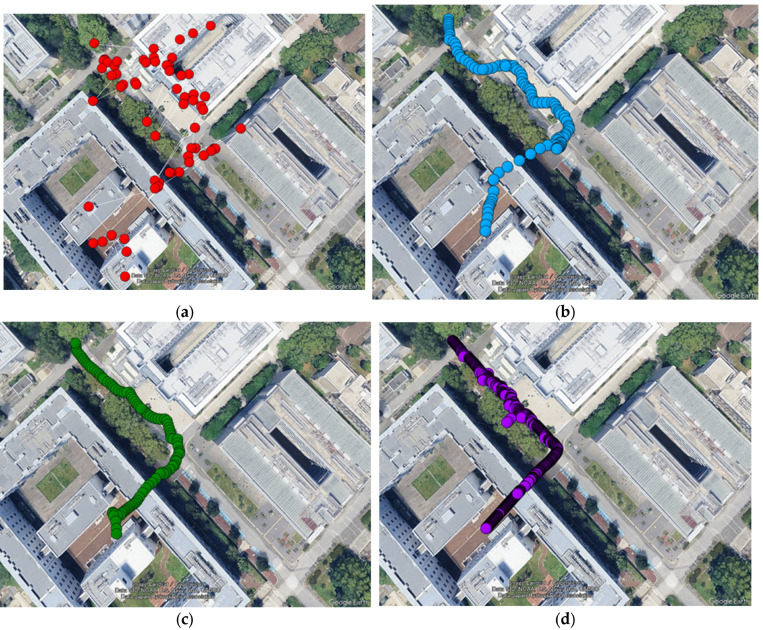
Plot of the estimated trajectory. (**a**) GNSS-only; (**b**) TC FGO-based GNSS/IMU integrated navigation; (**c**) GNSS/IMU integrated navigation using TC FGO with integrated NLOS detection; (**d**) reference values obtained from the GNSS-RTK solution. (Image: Google Earth Pro, © 2026 Google.)

Figure 9 shows the estimated trajectories for each method plotted on the map.

In standalone GNSS positioning, the trajectory showed significant variation throughout the entire period, with sections deviating substantially from the road also observed. This can be attributed to the elongation of pseudorange caused by receiving Non-Line-of-Sight (NLOS) signals, which distorted the geometric positioning solution. Furthermore, in sections passing beneath buildings, the positioning solution was partially interrupted. These findings also indicate that the experimental environment presented poor reception conditions for GNSS satellite signals.

In the implementation of GNSS/IMU integrated navigation using TC FGO, the trajectory became smoother compared to GNSS-only positioning, and irregular variations were suppressed by constraints from the IMU and motion model. However, when passing beneath buildings, a gradual drift in the trajectory was observed. This is thought to be partly caused by assigning a large weight to NLOS signals with high signal strength, thereby introducing their error during optimization.

Finally, it was confirmed that the trajectories generated by the proposed method not only achieve smooth trajectories but also closely follow the reference values without deviation, even in sections passing beneath buildings. Even in poor reception environments with numerous NLOS satellites, stable positioning was maintained by dynamically disabling them and combining reliable satellite signals with IMU information.

**Figure 10:**
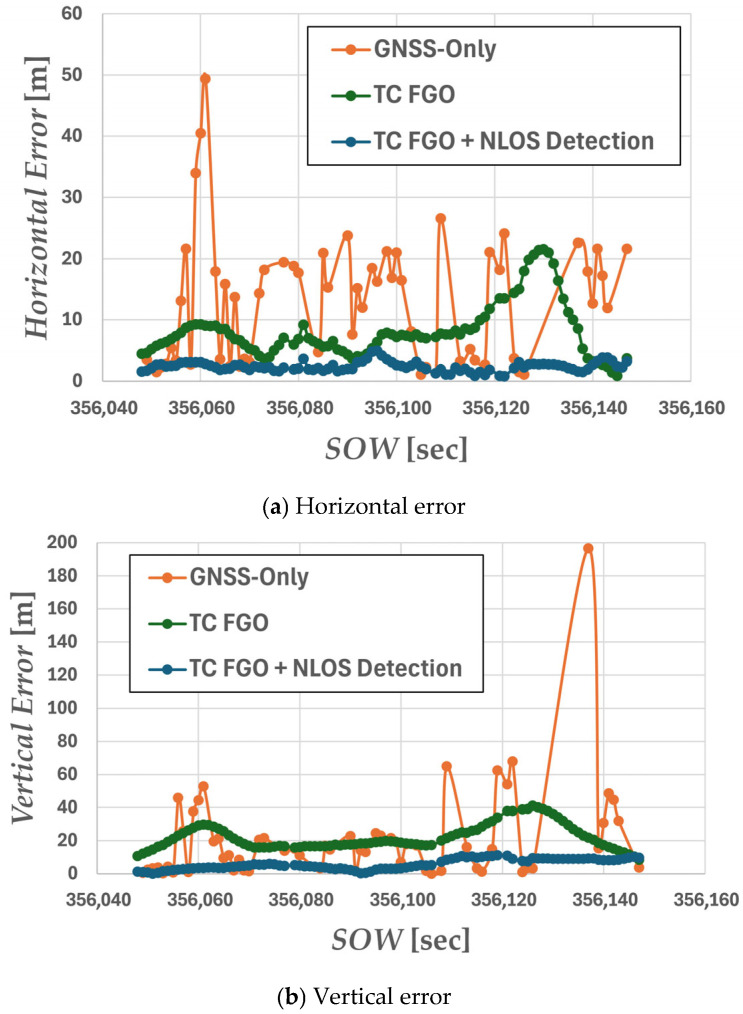
Plot of positioning error. (**a**) Horizontal error; (**b**) vertical error. The orange plot shows the positioning error for GNSS-only positioning, the green plot shows the positioning error for TC FGO-Based GNSS/IMU integrated navigation, and the blue plot shows the positioning error for GNSS/IMU integrated navigation using TC FGO with integrated NLOS detection.

Next, the positioning errors of each method were evaluated using quantitative metrics. Figure 10a,b show the temporal variations in horizontal and vertical errors, respectively. Table 2 presents the RMSE and maximum error for the entire experimental period.

Compared to standalone GNSS positioning, the methods using TC FGO demonstrated significant improvements in positioning accuracy across all cases. Notably, the proposed method achieved a 84.8% improvement in horizontal RMSE compared to GNSS Only. Furthermore, the proposed method exhibited a marked reduction in maximum error, confirming its high robustness to NLOS signals as a navigation system.

Next, to verify the robustness of the estimation in the proposed method, we analyzed the distribution of the posterior residuals, which are the pseudorange residuals after optimization. Here, the residual $r_{k,i}$ is the difference between the observed pseudorange $\rho_{k,i}$ and the theoretical distance $h_{GNSS,TC,i}\left(x_{k}\right)$ calculated from the optimized state estimate ${\hat{x}}_{k}$, expressed as in Equation (24).

(24) $$r_{k,i}=\rho_{k,i}-h_{GNSS,TC,i}\left({\hat{x}}_{k}\right),$$

**Figure 11:**
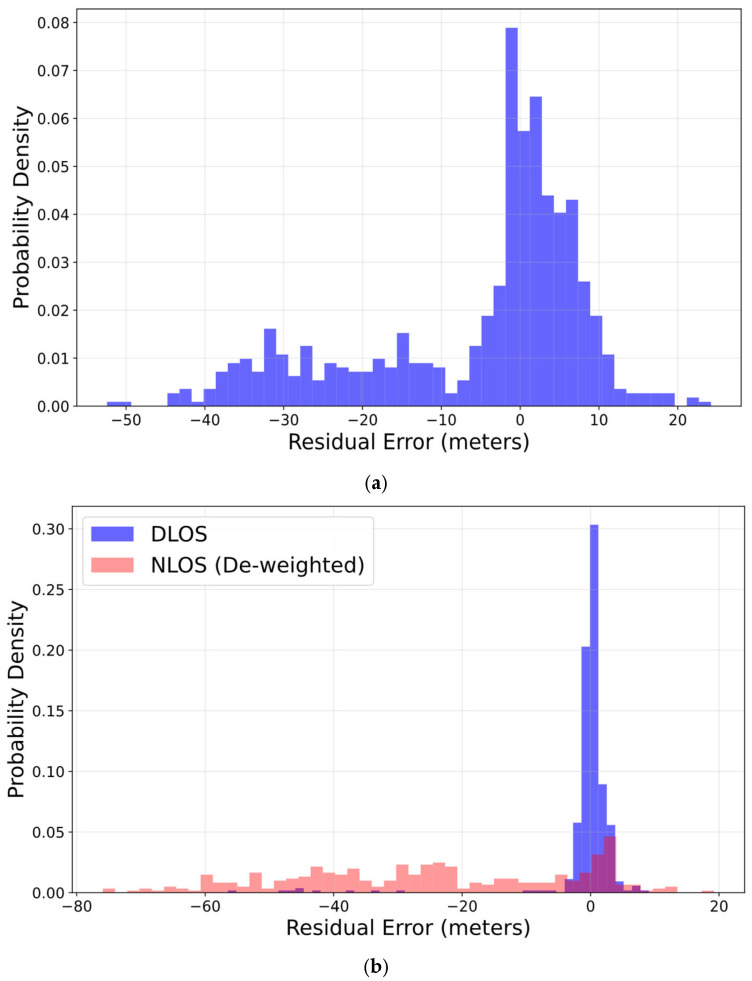
Posterior residual probability density of pseudorange. (**a**) probability density for TC FGO-based GNSS/IMU integrated navigation; (**b**) probability density for GNSS/IMU integrated navigation using TC FGO with integrated NLOS detection. The distribution indicated in red is effectively invalidated.

Figure 11a,b show the post-processing residual probability density for all satellites in this experiment. Figure 11a depicts the distribution when implementing GNSS/IMU integrated navigation using TC FGO, while Figure 11b shows the distribution for the proposed method. In Figure 11b, blue represents the pseudorange residuals of satellites that were not judged as NLOS and were used in optimization, while red represents the pseudorange residuals of satellites that were judged as NLOS signals and assigned extremely large variances. Figure 11a shows that the distribution in the conventional TC FGO is broad. In conventional TC FGO, a broad distribution pattern can be observed. This is thought to stem from the fact that conventional TC FGO utilizes all observed satellite signals for optimization, erroneously assigning small variance to NLOS signals despite their actual significant bias. The solver attempts to minimize errors caused by these NLOS signals, causing the estimated position to deviate from the true value.

In contrast, the residual probability density of the proposed method shows residuals from satellites used in optimization forming a sharp, zero-centered normal distribution. This indicates the estimated position maintains high consistency with the group of normal satellites. Simultaneously, the probability density shows data points with large residuals spread out. These are satellites judged as NLOS, assigned extremely large variances, and effectively rendered invalid. This indicates that satellite signals judged as NLOS actually contain large biases and are highly likely to be NLOS signals. In the proposed method, even if such large residuals remain, assigning them extremely large variances prevents the solver from attempting to forcefully reduce errors caused by NLOS signals. Instead, it ignores them as outliers. Consequently, the estimated position is determined solely by reliable satellites, unaffected by NLOS signals, leading to high-precision positioning.

Next, we considered maintaining accuracy in environments where GNSS signals are obstructed. In urban environments, situations where GNSS signals are completely blocked or severely degraded are anticipated, such as under elevated structures, in tunnels, or when entering indoor parking garages. As shown in Figure 7a, the experiments in this study also included driving sections passing under buildings, meaning they encompassed GNSS signal-blocked environments. In such environments, since position estimation continues relying on acceleration information from the IMU, it is crucial to correctly estimate the position during sections where GNSS signals are receivable. Therefore, in this study, we varied the time of acquiring pre-data before passing under the building to verify its impact on positioning accuracy beneath the buildings.

In this verification, the amount of prior data provided to the system was altered by adjusting the start time of the optimization process in the proposed GNSS/IMU integrated navigation method using TC FGO with integrated NLOS detection. Specifically, the time $t_{entry}$ when the rover entered beneath the building was defined, and the analysis start time $t_{start}$ was set as shown in Equation (25).

(25) $$t_{start}=t_{entry}-∆t_{warmup},$$

**Figure 12:**
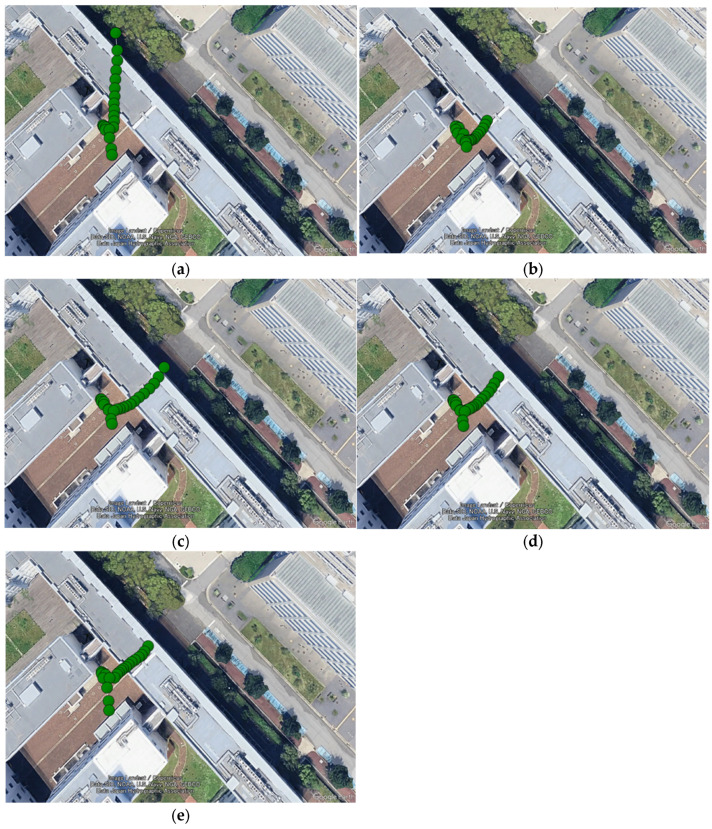
Plot of the estimated trajectory beneath the building. (**a**) $∆t_{warmup}=0$; (**b**) $∆t_{warmup}=2$; (**c**) $∆t_{warmup}=5$; (**d**) $∆t_{warmup}=10$; (**e**) $∆t_{warmup}=30$. (Image: Google Earth Pro, © 2026 Google).

**Figure 13:**
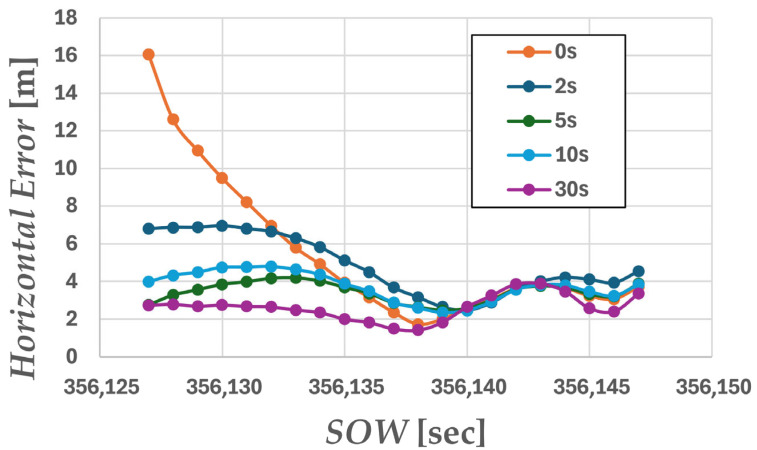
Plot of horizontal error beneath the building.

**Figure 14:**
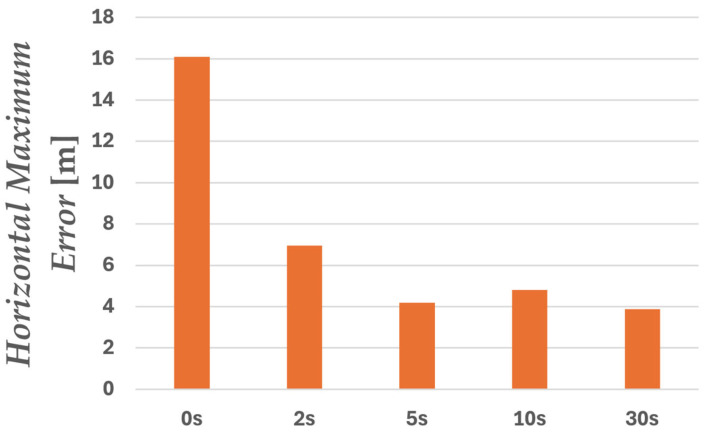
Maximum horizontal error beneath the building.

Here, $∆t_{warmup}$ represents the warm-up time from the start of analysis to entry, and five cases of $∆t_{warmup}=\left\{0,\;2,\;5,\;10,\;30\right\}$ were verified. Figure 12 shows the plots of the estimated trajectories on the map for each warm-up time during the shielded interval. Note that the analysis range includes the portion after passing beneath the building. Figure 13 shows the horizontal position error over time after entering beneath the building, while Figure 14 compares the maximum errors. Table 3 lists the RMSE and maximum error values. When the warm-up time was 0 s, the estimated trajectory deviated significantly from the actual path, with the error increasing sharply immediately after the start. This is thought to be caused by optimization being performed without correctly identifying the receiver’s position due to insufficient prior observation time. Similarly, solutions deviating from the travel path were obtained for 2 s. On the other hand, for 5 s, 10 s, and 30 s, the increase in error was suppressed. This is thought to be because the receiver’s correct position could be estimated before entry, allowing accurate inertial navigation to be maintained even within the obstructed section. Furthermore, the maximum error was also significantly suppressed for the 5 s, 10 s, and 30 s cases, indicating that the presence or absence of a pre-warm-up period affects positioning stability in the obstructed section. Although the 30 s case achieved the greatest suppression of maximum error, the 5 s and 10 s cases also showed nearly comparable error levels. Therefore, depending on operational performance requirements, it is considered that positioning can be maintained without significant error with a minimum warm-up time of 5 s.

Based on the above, the proposed method in this study can be said to maintain stable positioning even in temporary GNSS signal-blocked sections, such as under elevated structures or in tunnels, provided it has a few seconds of warm-up time. Furthermore, it is considered to possess robustness that enables it to immediately output high-precision positioning solutions even in situations where the system suddenly undergoes a cold start due to positioning equipment malfunction.

## 5. Conclusions

This study aimed to realize high-precision and highly reliable positioning using low-cost, compact equipment in severe urban multipath environments. To this end, we proposed a novel tightly coupled GNSS/IMU integrated navigation system based on factor graph optimization (FGO), augmented by a machine learning-based NLOS detection module. By utilizing the signal strength difference between RHCP and LHCP components from a dual-polarization antenna, we successfully trained a random forest classifier that identifies NLOS signals with a high accuracy of 0.89. This confirms the efficacy of exploiting physical polarization characteristics for signal classification in urban areas.

However, the current classifier uses only three features, making it quite simple. In the future, we plan to improve classification performance by exploring and utilizing a wider range of features. Furthermore, regarding the acquisition of training data, this study collected data from only two locations, resulting in a lack of data diversity. To ensure the model can adapt not only to the experimental environment used in this study but to any environment, a key challenge is to utilize training data collected from a wide range of environments, including other cities.

Unlike the conventional Extended Kalman Filter (EKF), the FGO framework preserves historical observation data and optimizes the entire state trajectory simultaneously. By dynamically assigning extremely large variances to the pseudorange factors of satellites identified as NLOS by the ML model, the proposed method effectively nullifies the detrimental impact of non-Gaussian outliers while maintaining the structural integrity of the graph.

Kinematic experiments in real-world urban canyons, including sections with complete GNSS signal occlusion (underneath buildings), demonstrated that the proposed method reduced the horizontal RMSE by 84.8% compared to standalone GNSS positioning. Furthermore, it significantly outperformed conventional TC FGO by strictly suppressing the maximum error, thereby proving its exceptional robustness against NLOS signals. We also investigated the system’s performance during GNSS outages and found that a brief warm-up period of approximately 5 s prior to signal loss is sufficient to constrain the maximum drift error to roughly 4 m during the occlusion. This highlights the system’s practical reliability for scenarios involving temporary blockages or immediate cold starts.

Despite these promising results, the current implementation relies on post-processing. Transitioning to a real-time system is a crucial next step for practical deployment. Introducing incremental optimization algorithms, such as iSAM2 [17], will be essential to achieve real-time, high-precision positioning with reduced computational overhead. In addition, rather than applying a fixed large variance based on the NLOS determination results, the use of loss functions such as the Huber function or the Cauchy function is expected to further enhance the system’s robustness. Furthermore, integrating additional sensors, such as LiDAR or vision cameras [18], into the factor graph framework could further enhance the resilience of the self-localization system. Ultimately, the proposed tightly coupled architecture—fusing ML-based physical signal classification with FGO—offers a scalable and robust solution that can significantly contribute to the safety and reliability of next-generation autonomous mobility, including self-driving vehicles and UAVs [19].

## Figures and Tables

**Table 1:** Three features used for classification.

Features	Symbols	Units
Satellite Elevation Angle	EL	Deg
Carrier-to-Noise-Density Ratio	$C/N_{0}$	dB-Hz
Standard Deviation of Carrier-to-Noise-Density Ratio	$C/N_{0_{std}}$	dB-Hz

**Table 2:** Error comparison between GNSS-only, TC FGO, and TC FGO + NLOS detection.

Methods	RMSE [m]		Maximum Error [m]	
	Horizontal	Vertical	Horizontal	Vertical
GNSS-Only	16.5	35.5	49.4	196.5
TC FGO	9.16	23.2	21.6	41.0
TC FGO + NLOS Detection	2.50	6.50	4.91	11.2

**Table 3:** Comparison of error between $∆t_{warmup}$.

$∆t_{warmup}$	Horizontal RMSE [m]	Horizontal Maximum Error [m]
0 s	6.65	16.1
2 s	5.09	6.96
5 s	3.46	4.19
10 s	3.81	4.80
30 s	2.71	3.88

## Data Availability

The data supporting the findings of this study are not publicly available because they are part of an internal university research project and are subject to institutional restrictions.

## Abbreviations

The following abbreviations are used in this manuscript:

AHRS	Attitude Heading Reference System
GNSS-RTK	GNSS-Real Time Kinematic
